# Between funder requirements and ‘jobbing scientists’: the evolution of patient and public involvement in a mental health biomedical research centre - a qualitative study

**DOI:** 10.1186/s40900-020-00185-7

**Published:** 2020-04-17

**Authors:** Joanne Evans, Stan (Constantina) Papoulias

**Affiliations:** 1 Department of Psychology, King’s College London, London, SE5 8AF UK; 2 Department of Health Service and Population Research, King’s College London, London, SE5 8AF UK

**Keywords:** Patient and public involvement, User led research, Biomedical research, Knowledge translation, Mental health

## Abstract

**Background:**

In the UK, there has been a strong drive towards patient and public involvement (PPI) in health research. Its benefits include improvements in the quality, relevance and acceptability of research, and empowerment, self-respect and value for service users. Organisational context can significantly influence the operationalisation of PPI. Research has highlighted power asymmetries between clinicians, researchers and service users. A resistance to power sharing, tokenism and assimilation into the existing culture suggest that a consultative, technocratic form of PPI is operating within health research settings. The aim of the study was to explore the development of PPI within a London based mental health biomedical research centre (BRC) over a period of 10 years from its inception.

**Methods:**

This qualitative study compared data from 52 organisational documents and 16 semi-structured interviews with staff and service users associated with PPI within the Maudsley BRC. The data were analysed using inductive thematic analysis. Study design, data collection, analysis and write up were conducted by service user researchers.

**Results:**

Our analysis showed a picture of increasing activity and acceptance of PPI, its alignment with the broader BRC research agenda, progressive involvement of service users in governance, and the development of a collaborative culture in research processes. The presence of salaried service user researchers in the organisation was key to this progress. However, PPI remained localised and under resourced and there was a reluctance to change working practices which resulted in perceptions of tokenism. Service users faced conflicting expectations and were expected to assimilate rather than challenge the organisation’s ‘biomedical agenda’.

**Conclusions:**

Service user researchers may play a key role in establishing PPI in a scientific, hierarchical research environment. Adoption of a more democratic approach to involvement would build on the good work already being done and help to transform the culture and research processes. However, such an adoption requires considerable changes to the funding and policy environment orienting health research.

## Plain English summary

### Background

In the UK, patients and the public have increasingly been involved in National Health Service research. Patient and public involvement (PPI) improves the quality of research and has a positive impact on those involved. The structure and culture of an organisation can influence how much involvement service users have. Research has shown that power hierarchies may be difficult to change, and service users report tokenism and having to fit into the existing culture, which results in a limited form of patient and public involvement. We were interested in the level of involvement service users had in a mental health biomedical research centre (BRC) over a period of 10 years.

### Methods

We analysed 52 organisational documents and interviewed 16 staff and service users connected to patient and public involvement. Service user researchers conducted the whole study.

### Results

Over time there was an increase in user involvement, including participation of service users in the governance of the organisation and more work in PPI to advance the BRC’s research priorities. This had many organisational and personal benefits. The presence of salaried service user researchers played an important role in this increase. However, a power hierarchy remained, PPI did not transform research priorities and working practices were difficult to challenge. Overall service users were expected to fit in rather than transform the culture and priorities of the organisation.

### Conclusions

Despite much progress, we have highlighted some of the difficulties associated with establishing user involvement in a scientific, hierarchical research setting. The presence of salaried service users is important for change to happen, but the funding and policy priorities that drive the organisation can limit this.

## Background

In the UK, there has been a strong drive towards patient and public involvement (PPI) in health research and a premium is placed on full involvement and equal partnership in the generation, adoption and dissemination of research knowledge [1]. PPI is required by major funders such as the National Institute for Health Research (NIHR) as a condition of funding and there has been a corresponding increase in PPI activity. This article considers the evolution of PPI in one prominent, NIHR-funded site for mental health research to analyse some of the openings and challenges associated with establishing and embedding service user involvement in a scientific and hierarchical research setting.

The organisational and personal benefits of PPI in health research are well documented. They include improvements in the quality, relevance and acceptability of research [2–4] increases in ethical practice [5, 6] raised awareness and acceptance of research among the research community [7] as well as empowerment, self-respect and value for service users [8, 9]. Furthermore, increasing attention is being paid to the role and function of PPI in research governance [10]. Nevertheless, there is considerable evidence that despite funders’ insistence on the involvement of service users and members of the public in health research, the power dynamic between healthcare professionals, researchers and service users has changed little. Resistance to power sharing is common, and scientific knowledge is routinely privileged over experiential and other forms of knowledge [11–14]. In this context there are indications that involvement practice remains tokenistic [15] and underpinned by a technocratic rather than a democratic rationale. This means that involvement of patients and the public is researcher rather than user-led and is sought in the hope that it may improve research quality and treatment cost efficiency rather than challenge existing power structures and engage citizens in decision making [16, 17]. Overall, an ongoing marginalisation or exclusion of dissenting voices in health research settings has been identified [18]. This marginalisation may be particularly marked in mental health research since healthcare professionals who may be conducting the research are those who can also make decisions over service users’ mental capacity, compulsory treatment and detainment [9, 19–21].

Finally, difficulties in accepting meaningful PPI in health research may also relate to the organisational cultures of UK higher education. Here, intensification of a performance management culture, characterised by a drive for efficiency, metricisation and increasing emphasis on ‘impact’ as measured by highly cited publications, can also affect how PPI is enacted within academic research environments [22].

In England the organisational space of Biomedical Research Centres (BRCs) provides an important case study in testing these arguments. BRCs are partnerships between universities and NHS trusts funded by the National Institute for Health Research. Their objective is to facilitate translational research between basic science, clinical practice and patient benefit [23]. BRCs were initiated in 2007 as five-year grants and currently there are 20 active across England, several of which focus on specific health areas (e.g. cancer, ageing, mental health). As BRCs command a significant portion of NIHR funds, they are expected to play a leadership role in driving PPI forward in the context of maximising patient benefit [1]. Yet service user involvement is particularly challenging in this research environment: research translation from basic science to clinical application and patient benefit is conventionally visualised as a linear process/pipeline, beginning in the laboratory and ending in clinical interventions. Within this model, early stages are defined through scientific expertise alone, while service user contributions are generally seen as relevant only in later stages where they can inform participant recruitment, dissemination and adoption of interventions [24] Even as more recent work – especially the emergence of ‘integrated knowledge translation’ models – is challenging these assumptions, there is still considerable resistance to the idea that non-specialists and service users in particular can inform the direction of lab-based or early clinical work [25–28]. Therefore, understanding the extent to which these organisations have operationalised involvement, the challenges they have encountered in doing so and possible solutions, is important in addressing the scope, applicability and effect of patient and public involvement practice in health research. For our study we focus on the Maudsley BRC: a partnership between South London and Maudsley (SLaM) NHS Foundation Trust and the Institute of Psychiatry, Psychology & Neuroscience at King’s College London. Our aim is to explore the development of PPI within the Maudsley BRC and to understand how and to what end PPI is operationalised in this institutional space. Since the Maudsley BRC conducts research in mental health, our case study provides an opportunity to investigate some of the specific challenges relating to the partnership between researchers and service users in this area.

## Method

### Design

This is a retrospective qualitative study covering a period of 10 years from the Maudsley BRC’s inception. In order to understand how and to what end PPI was operationalised, we compared the overviews of PPI in formal proposals and reports intended for the funder, with people’s experiences of progress made and challenges encountered as PPI evolved over the BRC’s tenure. We assimilated data from 52 documents and 16 interviews. Documents included: nine outward/funder-facing (proposals/bids and annual reports, websites and published articles) and 43 organisational/inward-facing documents (minutes, meeting agendas, internal reports). For the 16 interviews we used purposive sampling to identify people in key positions in the organisation, including those who worked in PPI: clinical academics, those in PPI co-ordinating roles, as well as service users involved in BRC work [29]. We also used snowball sampling (by following some participants’ own suggestions) as this allowed us to work with local knowledge more effectively [30]. Since we were interested in tracking the development of PPI in the BRC, we sought to interview individuals who had been involved in earlier iterations, including some who were no longer working in this context.

### Procedures

Our study was undertaken between March 2016 and March 2017, towards the end of the second and at the early phases of the third iteration of the Maudsley BRC. The study was announced at BRC executive meetings, meetings of service user advisory groups and through BRC communications. Information sheets with a detailed description of the study were provided. All participants gave written informed consent and understood that they were free to withdraw their data at any time. The study received approval by the Research Ethics Committee of King’s College London (ref number LRS-15/16–2318).

Sixteen people were interviewed - six men and 10 women. Seven were non-service user staff in academic, clinical academic and non-academic management roles (referred to in this paper as Academic/Professional or AP). Four were salaried service user researchers/academics, researchers with lived experience of mental distress who are staff in the university. They mostly occupy fixed term posts, are funded externally and work on mental health research (referred to in this paper as SR). Finally, five were service user advisors (service users working in an advisory capacity who are not staff in the university, identified as SA). Most user researchers and user advisors had been working with the BRC between one and 8 years at the time of the interview. Most academic/professionals were permanent staff at the university.

Interviews were semi structured and conducted without reference to funder facing documents in order to avoid raising expectations or guiding participants’ responses. Topic guides covered understandings of PPI, the range and level of involvement in the BRC, whether PPI or their role had changed over time, some of the benefits and challenges of service user involvement and suggestions for the future. Interview duration ranged from 40 to 60 min with one interview lasting 20 min. All interviews were digitally recorded and transcribed verbatim.

### Analysis

Documents were analysed descriptively in order to establish a timeline and record of the development and implementation of PPI for the purposes of triangulation. Funder facing documents were also submitted to a rapid thematic analysis which was deliberately deductive and topic based [31] in order to identify key aspects of the role and function of PPI in the organisational structure.

Interview transcripts were analysed by two service user researchers initially using an inductive or semantic thematic analysis approach (that is, a data-driven approach in which the interviewees’ statements are used to drive the codes and themes) [32, 33]. However, the analysis also included deductive elements: it was theory-driven in part, informed by particular debates on the technocratic vs democratic rationales mobilising PPI and the power dynamic specific to PPI in mental health research. The first author read the interview transcripts several times, familiarised themselves with the data and proceeded to generate an initial coding frame. The codes were then grouped in a provisional thematic patterning which was reviewed by both authors to ensure that it was dependable and confirmable and that the data supported each provisional theme [34, 35]. During this process we continually adjusted the thematic frame, revisited themes and modified our focus accordingly. At this point both authors proceeded to check how the themes produced in the analysis of the interviews related to the role and function of PPI presented in the funder facing documents. In order to strengthen the comparison between funder facing documents and interview reports, both thematic trees were adjusted to answer to each other wherever possible (see Table 1). Finally our analytical process and our findings, including the thematic trees, were presented to the panel of service user advisors working in the BRC to check the credibility of our interpretation [34].

**Table 1:** Key themes

**Funder Facing Documents**	**Interviews and internal documents**
1) **Progressive embedding of PPI in research through dedicated personnel**	• Increasing momentum in PPI in research • Embedding PPI with limited capacity: “a thin resource that has to be targeted”
2) **Innovative methods aligning PPI to BRC research agenda**	• PPI can improve research design in support of the BRC’s objectives • The “biomedical agenda” shapes and constrains involvement
3) **Progressive embedding of PPI in governance**	• The pull of institutional inertia
	4) **Recognition of mental health service users as research partners** • Collaboration legitimises mental health service user voice for academic/professionals • Limits to recognition: selective inclusion and the paradox of the insider/outsider

For the purposes of this paper, we have concentrated on a comparison between the presentation of PPI in the initial proposals (bids) for the three iterations of the BRC and the interviews. We will also be referring to other documents: minutes of the executive board and other meetings and a published article on the Research Priority setting exercise (2014–2016).

In our presentation of interviewees’ responses we have used the three categories AP, SR and SA and have not specified roles or demographics further (for example we do not make a distinction between programme managers and academics). This is because the number of interviewees is relatively small and more detailed descriptions would compromise anonymity.

## Results

In analysing the three BRC funding bids, we identified three major themes/topics which created a picture of the role and progress of PPI across the life of the BRC. We then distributed the themes generated in the analysis of the interviews and organisational documents, so that they corresponded to those three over-arching theme/topics. We also identified a fourth overarching theme/topic unique to the interviews: the recognition of mental health service users’ voice in research partnerships with two subthemes on the process and limits of this recognition (Table 1).

### Applications to NIHR (bids): 2006; 2011; 2016

PPI was presented as a particular strength of this BRC across all funder facing documents.

1. Progressive embedding of PPI in research through dedicated personnel

Proposals and annual reports claim that the involvement of service users and carers is becoming central to the BRC’s work overtime: *“patient involvement is not tokenistic but is a core element throughout the BRC” (2016 renewal proposal)*. Key to this claim was the university’s hosting of two innovative hubs for service user involvement which predated the inception of the BRC: the Service User Research Enterprise (SURE) and the Mental Health Research Network (MHRN). SURE is a research unit established in 2001, co-led by a user academic and staffed primarily by service user researchers, while the MHRN (2003–2014), was a ‘topic specific’ arm of the broader, England-wide clinical research network (CRN), a key part of NIHR infrastructure set up to support planning and undertaking of clinical trials and large-scale studies. The MHRN had a marked orientation towards PPI and employed researchers with a history of service use. Resources and expertise from both SURE and the MHRN were incorporated into the initial BRC to constitute a dedicated involvement ‘theme’ or research group, which was present across all BRC renewals: this ‘theme’ would both model collaborative research as well as facilitate the embedding of PPI across the BRC. The bids provided ample examples of PPI activity: collaboratively developed patient-reported outcome measures (PROMS) adopted nationally and internationally (all three bids); initiatives relating to infrastructure, such as the Clinical Records Interactive Search (CRIS), a powerful tool for searching de-identified electronic health records developed with patient consultation and overseen by a ‘*patient-led governance framework’* and a personal health record system, enabling service users to be *‘involved in their own care’* (MyHealthLocker) *(2016 Bid)*.

2. Innovative methods aligning PPI to the BRC research agenda

The mission of the BRC ‘theme’ dedicated to PPI was to support and strengthen the BRC research agenda. In the first proposal, a ‘Stakeholder Participation Research Theme’ supported the proposed ‘disorder focused’ main research themes by embedding service user advisory groups across the BRC, training service users in research skills and enabling a better understanding of the needs and ‘beliefs’ of service users and carers to improve research relevance (2006 bid). The second bid complemented the earlier focus on disorders with one on a diagnostic and technological infrastructure in the form of biomarker research and tools to enable mining of large clinical record datasets. Here, a ‘Patient and Carer Participation Theme’ was to play a key role in the development of this infrastructure and in ensuring that *“patient and carer [..] perspectives are integral to the science across the Centre”.* The third bid built on this infrastructure to support the development of “precision psychiatry” and to *“transform mental health treatment, delivery and prevention”* with a focus on early intervention. Here ‘Patient and Carer Involvement and Engagement’ was upgraded to one of four overarching themes/clusters signalling the “*critical importance of our partnership with patients and carers in the delivery of the proposed BRC’s objectives” (2016 Bid).* Notably, the third proposal also emphasised an 18-month research priority setting exercise involving local user and carer groups: this exercise featured prominently, with numerous examples of its influence to the proposed research projects and an overall claim that it had informed the BRC strategy and direction of travel.

3. Progressive embedding of service users and carers in BRC governance.

The first bid proposed service user representation on the Executive committee and the setting up of two service user advisory groups. The second bid would extend representation across the management structure and add a new patient reference group. The final bid (2016) described PPI as fully embedded, including strategic oversight, infrastructure to support research in the form of diverse advisory groups and links to public engagement. Furthermore, the BRC described itself as a leader and ambassador in the field of PPI: the *“only institution in the world with a critical mass of researchers with a key interest in collaborative and partnership research with mental health patients” (2016 Bid)*. It considered itself well placed to share best practice and become a national resource for other BRCs.

### Perceptions of patient and public involvement

#### 1a. Increasing momentum of PPI in research

Interviewees confirmed the narrative of progressive embedding of PPI across the three iterations of the BRC present in the proposals: *“it just feels like they are climbing all the time in terms of the development which is great” (SR1)*. Over the years, PPI had become *“much more developed and central to the role of the BRC.” (SA1)* Interviewees indicated that the influence of SURE was central to this development: *“I remember some of the early discussions where it was like well, you know, we’ll consult with service users, but [..] we’ll carry on … so having SURE there as this kind of constant presence that we were always going to continue making the arguments for why consultation was not enough, is absolutely crucial” (SR2)* Indeed the service user and carer priority setting exercise, which was specifically designed to inform the third BRC bid, was devised and facilitated by service user researchers and advisors and based on earlier work by SURE [36]. Academics described its results as *“absolutely embedded” (AP1)* in the 2016 bid and considered them to have had a *“significant influence” (AP6)* over the strategy and direction of funding into research valued by service users.

The contribution of people with lived experience working as salaried researchers was highlighted by user researchers and academics alike: *“so far we’ve always had service user researchers by hook or by crook. We will try again to make sure we have those people in the BRC” (AP4)*.

Furthermore, those tasked with PPI co-ordination felt that there was considerable buy-in from senior academics with a genuine respect and enthusiasm for involvement: *“if you just keep knocking on the door and just keep pushing in this direction ... it’ll work ... because the people are quite open minded and that comes from the top down” (SR3)*.

This observed evolution in PPI over the years was accompanied by a growing sense of shared responsibility. Staff responsible for PPI activity reported a shift in researchers’ attitudes: *“people are starting to think about how they do it and that’s really good to see. In the past it would have been, can you find me someone, can you do this?” (SR1)* Integral here was the early and ongoing implementation of PPI and the systematic measurement and monitoring of PPI progress and outcomes to ensure that *“it’s a reflective process all the time - to try and make it better for everybody” (AP4).*

#### 1b. Embedding PPI with limited capacity: “a thin resource that [] has to be targeted”

However, while PPI activity in the BRC increased over the years, much of the collaborative research remained localised. Interviewees stressed the intense labour required to embed PPI across the organisation through a shared set of expectations, actions and accountability: *“there’s definitely a need to keep the pressure on. You gotta keep holding people to account so they don’t forget” (SR3)*.

In this context, distributing responsibility for PPI across the BRC remained challenging: *“a lot of the themes had actually relatively few people on the ground to embed service user involvement [ …*] *I remember meeting with the researchers who were working on eating disorders and we had various ideas, but there was just no capacity” (SR2)*. Consequently, the same key people tended to be named on grants as responsible for PPI: *“it’s a thin resource that .... probably has to be targeted. That’s a sort of institutional challenge” (AP3).* In 2014 this resource became thinner still with the restructuring of the Clinical Research Network, which meant that the MHRN ceased to exist. The organisation had been staffed by service user researchers with strong connections with BRC members and input in project design. While the BRC was able to retain some of these in its various advisory fora and services, others left the organisation:

*“when all networks got amalgamated [ … ] there were all these claims about [there] being no job losses, things not changing, but of course everything changed and jobs went … that was really dire, [..] PPI fell completely by the way side with the CRN [yet] things were very well sustained here. … it was a real contrast.” (SA2)*

Finally, the inclusion of carers proved challenging, *“we don’t have such a well-developed system for family members to contribute.” (AP6)* While references to carers featured in organisational documents and in the name of the theme dedicated to PPI, (Patient and Carer Participation theme in the second BRC), interviewees felt that the BRC focused on service user rather than carer involvement. Attempts had been made to establish a carer’s group, but they had been unsuccessful:

*“I tried to sort of set one up and then eventually we kinda just integrated those people into other structures … … [embedding] carers is tricky because they definitely have less time.” (SR3)*

#### 2a. PPI can improve research design in support of the BRC’s objectives

The role of involvement in supporting the successful delivery of BRC objectives featured prominently in interviews. The input of service users in the development of CRIS, the clinical record mining tool, was singled out because of its usefulness in alleviating public fears over potentially controversial uses of records

*“we’ve been hugely successful in delivering [CRIS], and again that was primarily because we had service user input right from the start.” (AP5)*

*“The whole kind of national crisis over the … use of health records [for research], … made it really, really obvious why you needed to involve service users in a very serious way in the use of electronic health records.” (SR2)*

Overall, involving service users and carers was seen to legitimise and promote research within the wider community, thereby increasing study recruitment:

*“Again, it’s providing that validation and credibility, encouraging people who’ve been through the research process and know what to expect, to actually help promote research as a positive thing.” (AP5)*

The value of service user advisory groups was similarly defined in terms of their ability to improve research design *“researchers can go … to get advice and guidance in terms of design of their studies” (AP5)*

*“I think the fact that there is a queue of people waiting … suggests that it is useful for the organisation, you know that there is a need for it … . [we can highlight] problems that they might encounter before they happen.” (SA5)*

#### 2b. The “biomedical agenda” shapes and constrains involvement

However, while recognising how serving the BRC research needs has given credibility to PPI, some participants also reported that the organisation’s “biomedical agenda” restricted the forms involvement could take. They referred to the kinds of scientific models underpinning BRC funded research, for which PPI wasn’t considered a *“natural bedfellow” (SR3)*. In a context in which randomised control trials (RCTs) and systematic reviews constituted the strongest form of evidence, service user experiences were harder to legitimise:

*“I’m not sure that your jobbing scientists in the BRC are persuaded and really, those are the people who need to get the message” (SR4).*

Some participants felt that, in this context, demonstrating to *“jobbing scientists” (SR4)* how PPI might contribute to this work was a constant struggle: *“they’ll say you can’t involve people .... ‘cos it’s so early stage or it’s ... so abstract” (SR1)*.

Even when the importance of PPI was recognised, it was typically academics who decided its utility and role: *“you don’t need people to tell you how to design the study more scientifically rigorously. You need people to tell you how to design a study so it’s more feasible, acceptable” (AP4).*

Indeed, participants reported that during the first and second BRC, setting the research agenda remained the prerogative of academics for the most part:

*“[..] some themes, they decided what kinds of studies they were going to run, and they were gonna run them! You know, the fact that I came in and said well could we potentially open this out, well no because we’re going to run these” (SR2)*

Furthermore, researchers were seen as “*… condemned to a clinical agenda” (SR4)* which encouraged a tendency to *“become somewhat cellular in your thinking” (AP2).* This meant that, overall, researchers failed to consider a broader socio-economic contextualisation of mental ill health. While a number of studies did address social, psychological and economic factors in mental health problems, *“it was hard for that to be really visible in part because of the name, Biomedical Research Centre” (SR2)*.

The priority setting exercise was specifically designed to address these limitations, by incorporating into the third bid user and carer research priorities. An article presenting the results of the exercise noted the *“predominance of service delivery priorities over biomedical ones”* amongst user and carer groups and urged that *“a BRC in mental health should conduct translational research not just in biomedical, but also in social and psychological contexts.”* [37]

Indeed, user priorities tended to involve more practical, service delivery and socio-economic concerns: how to manage the cessation of medication, how to help people get help before a crisis happens, how insecurity in income and housing can affect mental health. Nevertheless, interviewees noted that, overall, user priorities identified in the exercise appeared to echo or at least not to challenge those of the BRC: *“there were aspects of the exercise which were just completely aligned with what we wanted to do anyway” (AP1)*. Moreover, this alignment arose at the end of a series of translations which, as interviewees also observed, had been necessary to render these priorities actionable within a BRC context:

*“the data in its raw form was kind of quite difficult to categorise, things that would fall into the effects of socioeconomic things. So obviously kind of trying to translate that into something****a bit more tangible within the context****was ......quite a task.” (SA2 – emphasis ours)*

That task involved for example reworking user concerns about medication side effects and polypharmacy into a call for precision medicine. Interviewees indicated that users’ priorities may have been considerably altered through this process: *“there are quite a lot of things which relate to early intervention, but probably the service users wouldn’t necessarily recognise them as such” (AP4).*

### 3a. Embedding PPI in BRC governance: the pull of institutional inertia

While interviewees confirmed that individuals’ commitment to PPI was growing, the broader organisational culture was slower to change. The efforts to embed PPI in BRC governance which gathered force during the second iteration of the BRC are instructive in this respect. Minutes from the 2014 Executive meetings indicate feverish activity after the funder demanded evidence of service user presence in BRC governance. Following deliberations between senior academics (including a senior user researcher) it was decided that service user advisors would attend meetings at the intermediate ‘cluster level’ (the four administrative units overseeing research and capacity building and reporting to the Executive). Furthermore, clusters were to give regular progress and impact reports of PPI, while deputy cluster leads were made responsible for setting this up. While service user advisors were allocated to cluster level meetings, updates from clusters remained erratic and inconsistent. The attempt to consolidate managerial support by giving deputy leads responsibility for PPI was abandoned after only two attempts due to non-attendance. Interviewees had very different responses to the effectiveness of this move: while some academic/professionals reported that they had found this allocation appropriate, user researchers and advisors indicated that it was unclear whether this was the right forum and whether service users did indeed have an input in decision making:

*clusters are where they’re deciding about the future spend of their money, whereas [in] the Executive board [..]I just think they would be bored out of their minds, coz there wouldn’t be anything for them to comment on (AP4)*

*it’s quite difficult to have any meaningful contribution because I think a lot of the decisions are not taken in [the cluster meetings] … it’s at project level, or programme level that the decisions are taken (SR4)*

*So, somebody was allocated to a cluster and then they … went to the cluster meetings and [ … ] that was where I started thinking, I’m not sure this is the right place for us to be having input (SA5)*

While one service user advisor did become involved in aspects of the third BRC bid, others gave examples of attending meetings where they had no specific function, where discussions were irrelevant to them or their views were not taken into account: *‘I had nothing to offer them, other than fulfilling the criteria of a service user being there’ (SA2).* A lack of clarity over the role and function of representatives in the clusters meant that their presence could be tokenistic, even a form of *‘political correctness’ (AP5).*

### 4. Recognition of mental health service users as research partners

#### 4a. Collaboration legitimises mental health service user voice for academic/professionals

Participants felt that researchers’ difficulty in seeing service users as equals with a valid contribution to shaping research is particularly pronounced in mental health. This reflected *“how people with mental health issues are perceived by people who are professionals in the field” (SA5)*. Service users observed that collaborative work required a shift in the habits which defined researchers’ professional status and expertise, particularly when these researchers were also clinicians: *‘we’re conscious that ... some of the researchers are not used to working with patients in this kind of way. This is a different role. We are generally people who have recovered and are able to reflect on our patient experience and then try and use that .... in a constructive way, which is very different to dealing with a patient who is currently in a crisis’ (SA5)*. Academic/professional participants reported that such a shift was indeed occurring: *‘we’ve come from ... saying yes we need to involve patients because they’re the subjects of our research to realising no, that’s not really good enough’ (AP6).* One service user advisor’s input was described as ‘*invaluable. It’s really changed ... how we communicate with each other......’cos we’re just so used to talking to each other in this coded language’ (AP2).* Service user interviewees also reported a reduction in inappropriate language over time, and increased use of lay summaries so that they were better informed.

Service user advisors emphasized a sense of fulfilment from being recognised as part of a research community. For some, involvement had a positive impact on confidence and was a step towards recovery: *‘coming to a place like this, you may be initially silent but eventually it’s part of the process that helps you to get back*’ *(SA3)*. Many spoke of a sense of empowerment, which they articulated as a recognition of the worth of service user experience by researchers. Those service user advisors who had been involved in the research priority setting exercise felt that their views *“really mattered” (SA3)* and had the potential to *“shape research” (SA2).* This increased sense of agency run counter to some service users’ experience of healthcare services: *‘feeling respected by people that have quite a lot of power [is] like the reverse..[of my experience of services].. it’s been helpful on a personal level’ (SA4)*. Some researchers too valued how working together could alter the dynamic between service users and researchers: *‘it’s about a collaboration and it’s about that collective approach from the very beginning’ (AP2).*

#### 4b. The limits of recognition: partial inclusion and the paradox of the insider/outsider

While service user participants recognised steps toward greater recognition of service users as partners in research over the years, they also spoke of being part of a rather anaemic or *“sanitised” (SR2)* version of service user involvement – users who were largely white, middle class and well educated, and were able to communicate effectively with academics.

*“what does it mean to be proud of having more people like me who have lots and lots of privileges, kind of representing service user involvement, when you actually still don’t have the voices of a whole massive range of people who are not represented in Biomedical Research Centres?” (SR2).*

Academic/professionals too spoke of valuing users with relevant knowledge and skills:

*“[name of service user advisor] had in a previous life a lot of that sort of strategic oversight, and so they brought that as well as the experience of the service user within the system. It was good” (AP2).*

Furthermore, some academic/professionals testified to an organisational reticence to involve service user researchers or advisors who could challenge the existing culture: “*investigators are frightened of .. conflict. In mental health there is … a history of ... the agenda not.being aligned [to] what service users want .... [but] what clinicians, academics often are preoccupied with” (AP1).*

Overall work with service users within the BRC involved a key paradox: academic fluency, while desired by academics, was also looked upon with suspicion, as though it could get in the way of service users’ ability of *“being expert in their own experience” (AP4)*

*“my only concern is .... to not have expert patients.... where they forget they're a patient and become a researcher” (AP4).*

The presence of salaried service users with an academic background put a strain on the very definition of PPI. As one senior participant wondered: *“here’s a tricky little thing. Is it still PPI when the service user is an academic?” (AP7)*. This double demand for service users to be able to navigate academic environments while also remaining outsiders to these could result in a paradox: *“Nobody can speak. You can’t speak if you’re too articulate and obviously if you’re not articulate you can’t speak either, so it’s a complete catch 22” (SR4).*

## Discussion

Between the first and third iteration of the BRC, PPI became increasingly embedded and working with service users and carers became more broadly acceptable. Participants’ perceptions and organisational documents alike confirmed the reported diversification and enrichment in PPI activities and evidenced considerable collaborative research work (notably in the establishment of the clinical records mining tool). Pre-existing research hubs (e.g. SURE and the MHRN) were fundamental to this process, as they embedded a host of service users with in-depth knowledge of research cultures within the BRC. Indeed the employment of salaried service user researchers attenuated one of the key difficulties in embedding PPI identified in the literature: typically ‘public contributors’ are not able to share day to day habits and rituals of research practice and therefore their opportunities to influence such practice are limited [24]. Additionally, work with service user advisors, the proliferation of local user groups (such as a young people’s group) and especially the priority setting exercise, all demonstrate an organisational willingness to engage with more diverse local voices and to open up the research agenda to local needs.

However, while practice diversified and collaboration became more acceptable, overall the model of PPI remained broadly technocratic and academic led: involvement was meant to serve rather than challenge the epistemological underpinnings of biomedical research and the broader institutional research agenda. For those ‘jobbing scientists’ who saw value in involvement, such value lay in its potential to expedite the delivery of BRC objectives: that is, PPI was perceived as a useful add-on to organisational culture and to business as usual, rather than as a practice with the potential to transform institutional habits and priorities. Salaried user researchers, with their immersion into such habits, exemplified the challenges to transformation: on the one hand salaried user researchers produced work that both adds to and contests institutional priorities [25–28]. On the other hand, insofar as user researchers are assimilated within the BRC and essential to its success, their presence may inadvertently perpetuate exclusions of less assimilable voices, perspectives or groups [21, 38].

Arguably the successful bid for the third round of NIHR funding incorporated local service user perspectives to a much greater degree than other organisations of this scope and size. However, it seems that through a process of translation and alignment, this incorporation continued to support and legitimise the broader directions of the organisation’s third iteration – in this sense engagement with local priorities was not designed to challenge the ‘biomedical definition of illness and its solutions’ but rather to supplement it [39].

In an influential paper on partnering with ‘patients’ [sic] in biomedical research, Caron-Flinterman and colleagues argue that the substantive embedding of end-users in research cannot be programmatically enforced but needs to be negotiated as a long-term transition process capable of engaging three levels of social organisation [40]:

1. A *micro level* of individuals whose actions establish “niches” of transformation
2. A *meso level* of networks which crystallise as “regimes” (established ways of ‘doing science’ and science communication, tacit rules of practice and hierarchies of evidence)
3. A *macro level* of material “landscapes” constituting shared cultures and paradigms

In the Maudsley BRC, SURE and the individuals and research projects developing around it constituted such ‘niches of transformation’ with the potential to challenge the ‘regime’ operating within the organisation/network. However, ‘regimes’ are not about individual actors (including those in positions of leadership) but about shared institutional habits through which expertise is established and networks stabilised. They are thus much slower to shift and, furthermore, they themselves orient research priorities and strategic decisions in accordance with a macro level of policy and funding. The relationship between the Maudsley BRC and such broader “landscapes” of health research and university funding is crucial here. NIHR calls shape the horizon of research and determine the kinds of expertise likely to receive funding. At the same time, funders currently require that institutions applying for funds *also* demonstrate appropriate involvement of service users and public members into this work. Yet crucially such impositions are not organically inter-related: funder/policy priorities for research are not *themselves* connected to service user priorities and, additionally, the time and resources necessary to embed PPI across an organisation or a programme of research are rarely taken into account in funder timelines, budget allocation or indeed in decisions to overhaul infrastructure (as seen in the abolition/absorption of the MHRN).

Thus, on the macro level of policy and funding, service users and the public are late arrivals to the scene of research: policy regarding their inclusion, while present, is generally disconnected from other funder priorities and broader agendas yet implicitly seen to support these The intensification of performance management in higher education forms another part of the same “landscape”: For many academics, doing PPI is not considered an appropriate use of ‘academic time’ – unless PPI related work can be repackaged as ‘outputs’ that is professional activities which directly impact career progression in a hyper-competitive, marketized arena [41]. In this sense, the requirement to work with service users and carers, especially if such work engages an emancipatory rather than a technocratic logic, can be an additional, competing demand which is likely to be resisted.

The difficulty embedding service users in Maudsley BRC governance can be grasped as an interplay of such macro level forces: since user representation in ‘cluster’ meetings was imposed in response to funder scrutiny, such representation was likely to be set up in a hurry and remain tokenistic rather than exert a transformational force to the institutional ‘regime’. Similarly, the requirement that certain academic leads attend additional service user meetings and deliver PPI reports to the Executive may have exacerbated unwelcome pressures for staff. When PPI is perceived as an imposition, academics may be less receptive to arguments in its favour [22, 42].

## Conclusion

While there has been considerable progress in embedding PPI in the Maudsley BRC between 2007 and 2017, our analysis suggests that this progress has not challenged the technocratic academic-led rationale for PPI within the organisation. We are certainly not the first to argue that PPI in research organisations is largely captive to technocratic or managerialist rationales – much empirical work on PPI comes to similar conclusions [39, 43–45] However, while such work tends to recommend a closer attention to the power dynamics between actors (researchers, managers, service users and others) we would additionally argue that in order for user involvement to have transformational force, equal attention needs to be paid on how broader socio-political structures define the scope and horizon of research and how funder priorities and policy directions constrain how user participation is both conceptualised and practiced [26]. In the UK, mental health service user researchers and academics have argued that, while PPI is increasingly gaining support by funders and policy makers, this support is constrained by ongoing structural barriers [18]. For example while forms of advisory PPI are encouraged, funders are unlikely to support user *led* research and knowledge production [46]; while many user organisations and groups, including those with expertise in PPI training, are also unable to secure funding.

Attending to the democratising potential of patient and public involvement in mental health research is increasingly important, particularly as ongoing austerity policies, welfare reforms and health service cuts in the UK continue to disproportionately affect mental health service users [47, 48]. In this context, Sarah Carr recently argued that user researchers working in higher education can adopt a bridging role, providing networking opportunities and access to resources for user groups [38]. The Maudsley BRC, with its history of employing service user researchers, its relationship to the user-led unit SURE at King’s College London and its ambition of forging ongoing links with local user groups may be well placed to support such initiatives. Whether it is able or willing to do so remains to be seen.

### Limitations

Our study was exploratory and did not attempt a formal evaluation of PPI. Furthermore, access and timeline considerations prevented us from working prospectively and we thus concentrated on developments during the first and second BRC iterations and early days of the third. As the Maudsley BRC specialises in mental health, some of the themes are likely to be influenced by issues around mental capacity, compulsory treatment and detainment which are not similarly applicable in physical health contexts. While participants discussed challenges to collaboration which were specific to mental health research, there was little overt reference to detainment and compulsory treatment. This reticence will need to be explored further in a subsequent study. Furthermore, while we initially planned to interview participants in key management positions across the BRC through purposive sampling, in the event, most of our participants who were not service user advisors or researchers were those with an avowed positive attitude towards involvement. This potential bias is encountered in several studies of this kind and may indicate that an engagement with PPI is still not considered legitimate use of ‘academic time’.

Finally, while this project was carried out by user researchers in its entirety, we were unable to work with user advisors (non-salaried service users). Increasingly the delivery of applied health research projects depends on research assistants whose contractual timelines and other obligations constrain capacity and do not allow for much flexibility in delivering a project, thus making a more collaborative approach more difficult to sustain.

## Data Availability

The datasets generated and analysed during the current study are available from the corresponding author on reasonable request.

## Abbreviations

BRC: Biomedical Research Centre
CRN: clinical research network
MHRN: mental health research network
NHS: National Health Service (United Kingdom)
NIHR: National Institute of Health Research (United Kingdom)
PPI: patient and public involvement
SUAG: service user advisory group
SURE: service user research enterprise (research unit at King’s College London)
UK: United Kingdom
AP: Academic/Professional
SA: Service User Advisor (service users having occasional contact with the organisation in an advisory capacity - usually in groups)
SR: Service User Researcher (salaried researchers with a history of service use)
